# UCHL5 controls β-catenin destruction complex function through Axin1 regulation

**DOI:** 10.1038/s41598-022-07642-1

**Published:** 2022-03-07

**Authors:** Wonhee Han, Youngmu Koo, Leila Chaieb, Byeong-Rak Keum, Jin-Kwan Han

**Affiliations:** 1 Department of Neurology, F. M. Kirby Neurobiology Center, Boston Children’s Hospital, Harvard Medical School, Boston, MA 02115 USA; 2 Department of Life Sciences, Pohang University of Science and Technology, 77 Cheongam-Ro, Nam-Gu, Pohang, Gyeongbuk 37673 Korea

**Keywords:** Cell biology, Molecular biology

## Abstract

Wnt/β-catenin signaling is crucially involved in many biological processes, from embryogenesis to cancer development. Hence, the complete understanding of its molecular mechanism has been the biggest challenge in the Wnt research field. Here, we identified ubiquitin C-terminal hydrolase like 5 (UCHL5), a deubiquitinating enzyme, as a novel negative regulator of Wnt signaling, upstream of β-catenin. The study further revealed that UCHL5 plays an important role in the β-catenin destruction complex, as it physically interacts with multiple domains of Axin1 protein. Our functional analyses also elucidated that UCHL5 is required for both the stabilization and the polymerization of Axin1 proteins. Interestingly, although these events are governed by deubiquitination in the DIX domain of Axin1 protein, they do not require the deubiquitinating activity of UCHL5. The study proposes a novel molecular mechanism of UCHL5 potentiating the functional activity of Axin1, a scaffolder of the β-catenin destruction complex.

## Introduction

Wnt signaling has a myriad of pivotal roles in numerous biological processes, such as embryogenesis, human congenital disorders, and cancer development^1^. The transduction of Wnt/β-catenin signaling pathway starts when a ternary complex called the Wnt signalosome forms at the cell surface^2^. The Wnt signalosome is comprised of a Wnt ligand, a receptor (Frizzled), and a co-receptor (LRP5/6)^2^. The Wnt signalosome amplifies signal activity by recruiting downstream effectors, including Dishevelled (Dvl), Axin, Casein kinase1 (CK1), and Glycogen synthase kinase3β (GSK3ß) to the plasma membrane^3^. Within the Wnt signalosome, the intracellular domain of LRP5/6 is phosphorylated by GSK3ß. This phosphorylated intracellular domain functions as a docking site for Axin, and inhibits the kinase activity of GSK3ß^2^. Therefore, the Wnt signalosome not only inhibits the β-catenin destruction complex, but also sequesters it from β-catenin^2,3^. Consequently, the free cytosolic β-catenin moves into the nucleus, and forms a transcriptional complex with T-cell factor/lymphoid enhancer factor (TCF/LEF) to induce Wnt target genes^4^.

β-catenin stabilization in the cytoplasm is predominantly determined by the β-catenin destruction complex. The β-catenin destruction complex consists of multiple proteins, including the scaffold protein Axin, Adenomatous polyposis coli (APC), and two serine/threonine Kinases, CK1 and GSK3. Cytoplasmic β-catenin is normally recruited to Axin in the inactive state of Wnt signaling, and is subsequently phosphorylated at the serine/threonine residues (CK1: Ser45, GSK3β: Ser 33, 37, and Thr 44) of β-catenin by CK1 and GSK3^3^. APC recognizes and sets the phosphorylated β-catenin free from Axin for subsequent ubiquitination and proteasome-dependent β-catenin degradation^3^. This process of β-catenin turnover is shut down upon Wnt signaling activation. Specifically, Axin is most affected by Wnt signaling activation, as it is recruited to the plasma membrane after losing its binding affinity to β-catenin^5^. Furthermore, Axin is destabilized under long-term activation of Wnt signaling^6^. Therefore, Axin is a key molecule determining the functional activity of the β-catenin destruction complex. Indeed, Axin is the concentration-limiting factor in the β-catenin destruction complex^3^. Therefore, Axin must be tightly and sophisticatedly regulated to prevent abnormal activation of Wnt signaling.

One of the representative regulatory mechanisms determining Axin activity is ubiquitin-mediated Axin regulation. Many E3 ligases and deubiquitinating enzymes have been reported. RNF146, a RING-domain E3 ligase, recognizes ADP-ribosylated Axin and mediates its protein degradation^7,8^. Another RING-domain E3 ligase, SIAH(SIAH1/2), outcompetes GSK3ß for Axin binding and mediates Axin degradation under the Wnt active condition^6^. Lastly, Smurf2, a HECT-type E3 ligase, has been reported to attenuate Axin stability^9^. As for deubiquitinating enzymes, proteomic analysis revealed that USP34, a member of the ubiquitin-specific protease family (USP), has a role in reversing the Tankyrase/RNF146-dependent ubiquitination of Axin^10^. Intriguingly, this USP34 acts as a positive regulator downstream of ß-catenin by accumulating Axin in the nucleus^10^. Recently, genome-wide CRISPR/CAS9 screening and chemical analyses newly identified another USP member, called USP7, that stabilizes Axin under the Wnt active condition^11^. Nevertheless, the molecular basis of Axin ubiquitination and regulation remains elusive.

The functional activity of Axin is also determined by its oligomeric state. The C-terminal DIX domain of Axin is essential for its hetero- and homo-polymerization^12–14^. Specifically, the homo-polymeric state of Axin represents its functional activity as a scaffolder of the ß-catenin destruction complex^12–14^. The importance of the DIX-mediated Axin polymerization can be demonstrated by the complete abolishment of Axin polymerization and its suppressive activity simply by DIX deficiency or through point mutations within the ß -strands of the DIX domain^12–14^. However, how this DIX-mediated Axin polymerization is stably maintained, and what molecule governs the polymerization, are still enigmatic.

Here, we suggest Ubiquitin C-terminal hydrolase like 5 (UCHL5) as a key molecule maintaining the functional activity of the ß-catenin destruction complex. UCHL5 is a deubiquitinating enzyme (DUB) belonging to ubiquitin C-terminal hydrolase (UCH) subfamily^15^. UCHL5 plays multiple roles in diverse cellular processes by interacting with various protein complexes, such as the proteasome, the chromatin remodeling complex (INO80), and transcription factors (E2F1)^16–18^. With this molecular basis, UCHL5 has been known not only to rescue the substrate proteins from proteasome-dependent degradation, but also to regulate gene transcription^16–18^. In this study, we discovered that UCHL5 plays a negative regulator role in Wnt signaling in various cancer cells. UCHL5 also plays part in the ß-catenin destruction complex by interacting with Axin1. More importantly, UCHL5 stabilizes Axin1 protein by blocking ubiquitination of Axin1 DIX domain, thereby sustaining the DIX-dependent Axin1 polymerization.

## Results

### UCHL5 negatively regulates Wnt signaling upstream of ß-catenin

**Figure 1:**
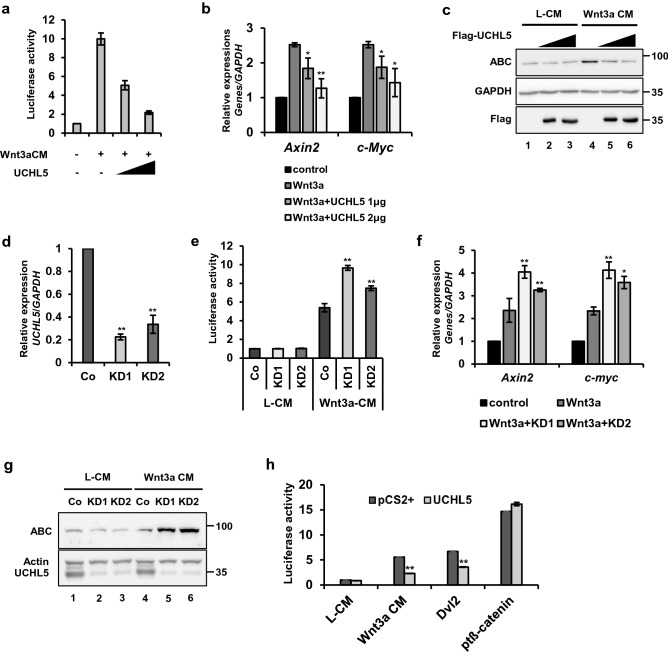
UCHL5 negatively regulates the Wnt/ß-catenin signaling pathway. (**a**) TOPflash assay using HeLa cells. Cells were transfected with the indicated plasmids (50 ng *TK-Renilla* reporter; 250 ng TOPFlash; 250 ng FOPFlash; 1 μg and 2 μg pCS2 + UCHL5). Then, Wn3a conditioned media (Wnt3a CM) was treated for 16 h. (**b**) Quantitative PCR (qPCR) analysis for Axin2, pCS2 + UCHL5 was introduced into HeLa and MCF7 cells. Then, Wnt3a CM was treated for 16 h. GAPDH was used for normalization. (**c**) Western blot analysis using HeLa cells. Flag-UCHL5 plasmids (2 μg and 4 μg) were transfected with empty vector. Transfected cells were treated with either L- cell control CM (L CM) or Wnt3a CM for 16 h. (**d**) qPCR analysis for UCHL5. Total RNAs were extracted from control (Co) HeLa and two different stable knockdown HeLa cells (KD1 and KD2). Samples were subjected to cDNA synthesis and qPCR analysis. GAPDH was used for normalization. (**e**) TOPflash assay using stable HeLa cells. Co, KD1, and KD2 HeLa cells were transfected with TOPflash (200 ng) and *TK-Renilla* reporter (50 ng). Then, cells were treated with Wnt3a CM for 16 h. (**f**) qPCR analysis for Axin2 and c-myc. Co, KD1, and KD2 HeLa cells were incubated with Wnt3a CM for 16 h. (**g**) Western blot analysis using Co and KD HeLa cells. Co, KD1 and KD2 HeLa cells were incubated with Wnt3a CM for 16 h. (**h**) TOPflash assay using Co and KD1 HeLa cells. Either pCS2 + or pCS2 + UCHL5 (1 μg) was co-transfected with TOPflash reporter (250 ng) and TK-Renilla reporter (50 ng) into cells. For Wnt stimulation, Wnt3a CM (for 16 h), Dvl plasmids (0.5 μg), and ptß-catenin plasmids (0.2 μg) were introduced. The data from TOPflash (a, e, h) and qPCR analyses (b, d, f) are displayed as means ± SD and show a representative of multiple independent experiments (n = 3 biological independent experiments). * *P* < 0.05, ** *P* < 0.005. from two-tailed unpaired t-test (**a**, **b**,**d**, **e**, **f**, **h**).

To determine the effect of UCHL5 on the Wnt signaling pathway, we employed a TOPFlash reporter containing repeated Wnt response elements (WREs) (Fig. 1a). We observed that the overexpression of UCHL5 (UCHL5 OE) negatively regulated luciferase activity in HeLa cells (Fig. 1a). Consistently, UCHL5 OE inhibited the expression of Wnt target genes, such as Axin2 and c-myc, as well as the level of active ß-catenin (ABC), in a dose-dependent manner (Fig. 1b,c lane 4,5,6). We also constructed stable HeLa cell lines (UCHL5 KD1 and KD2), which stably express two independent short hairpin RNAs (shRNAs) against UCHL5 messenger RNA (mRNA) (Fig. 1d). These two UCHL5 KD cells showed synergistic promotion of Wnt signaling activity with Wnt3a-conditioned media (Wnt3a CM). TOPFlash and qPCR results clearly showed further increase in the reporter activity and expression of Wnt target genes (c-myc and Axin2) under Wnt3a CM treatment (Fig. 1e,f). In addition, a higher protein level of ß-catenin was observed in UCHL5 KD cells compared to wild type cells after treatment of Wnt3a CM (Fig. 1g. lane 4, 5, 6). In contrast, there was no significant change in ß-catenin level under control CM-treated condition (Fig. 1c,g. lane 1 2 3). Next, we pinpointed the location of UCHL5 function within the cascade of Wnt/ß-catenin signaling pathway (Fig. 1h). UCHL5 OE significantly suppressed TOPFlash activities induced by Wnt3a and Dvl (Fig. 1h). However, UCHL5 OE had no effect on TOPFlash activity induced by constitutively active ß-catenin (pt ß-catenin) (Fig. 1h), which has resistance against GSK3ß-mediated phosphorylation and degradation by mutations at Ser 33, 37, and Thr 44. Taken together, the data revealed UCHL5 as a novel negative regulator of Wnt/ß-catenin signaling pathway upstream of ß-catenin.

### UCHL5 plays part in the ß-catenin destruction complex through Axin1

**Figure 2:**
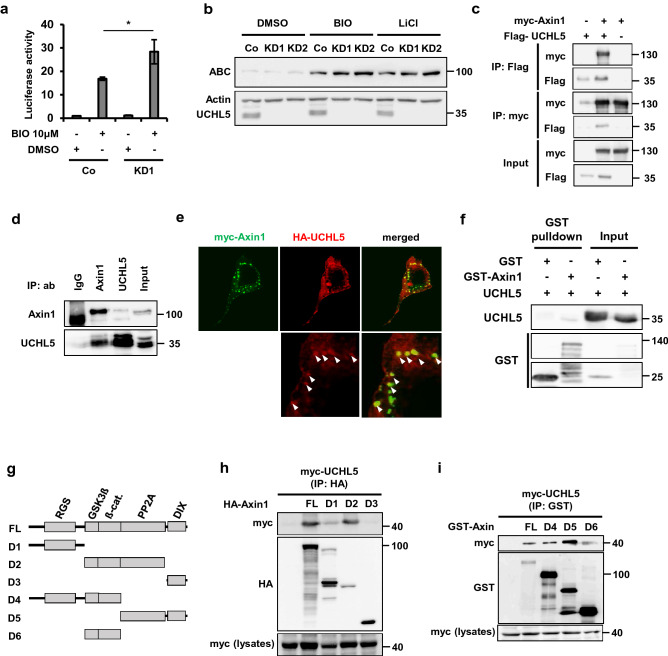
UCHL5 interacts with Axin1. (**a**) TOPflash assay using Co and KD1 HeLa cells. Co and KD1 HeLa cells were transfected with TOPflash (200 ng) and *TK-Renilla* reporter (50 ng). Then, cells were treated with BIO (1 μM) for 16 h. The data is displayed as means ± SD and show a representative of multiple independent experiments (n = 3 biological independent experiments). * *P* < 0.05. (**b**) Western blot analysis using Co and KD HeLa cells. BIO (2 μM) and LiCl (25 Mm) were added to Co, KD1 and KD2 HeLa cell culture media for 6 h. (**c**) Co-IP assay using HeLa cells. Indicated plasmids were introduced to cells (2 μg myc-Axin1; 2 μg Flag-UCHL5). The transfected cells were treated with MG132 (5 μM, 16 h) and then subjected to immunoprecipitation using anti-Flag and anti-myc. (**d**) Co-IP assay using HeLa cells. After treatment with MG132 (5 μM, 16 h), cells were lysed and precipitated with the indicated antibodies (anti-IgG, anti-Axin1, and anti-UCHL5). (**e**) Immunocytochemistry assay. HeLa cells were transfected with myc-Axin1 (200 ng) and HA-UCHL5 plasmids (200 ng). Transfected cells were then fixed and stained with anti-myc (green) and anti-HA (red). Scale bar represents 10 μm. (**f**). (**b**) In vitro binding assay using recombinant proteins. UCHL5 and GST-Axin1 proteins were incubated and then subjected to immunoblotting. (**g**) Diagram of a series of Axin1 deletion mutants used for IP assay. (**h**, **i**) IP assay using HeLa cells. Cells were transfected with Flag-UCHL5 (2 μg) and various truncated Axin1 mutants (2 μg D1-D6).

Given that UCHL5 negatively regulates Wnt/ß-catenin signaling pathway by destabilizing ß-catenin, we hypothesized that UCHL5 is functionally involved in the ß-catenin destruction complex. GSK3ß is a key component of the ß-catenin destruction complex, as it mediates ß-catenin turnover by promoting the integrity of the ß-catenin destruction complex and directly phosphorylating ß-catenin. Thus, we speculated that if UCHL5 regulates the ß-catenin destruction complex, the depletion of UCHL5 would further promote the activity of the Wnt signaling pathway. In order to inactivate the ß-catenin destruction complex, we inhibited GSK3ß by treating either BIO or LiCl, both GSK3ß inhibitors. As expected, UCHL5 KD led to a synergistic increase in TOPFlash activity with BIO (Fig. 2a). Consistent with the result of the TOPFlash assay, UCHL5 KD showed a relatively higher level of ß-catenin protein expression in comparison to WT cells under GSK3ß-inhibited conditions by both BIO and LiCl (Fig. 2b). The results supported the possibility that UCHL5 functions within the ß-catenin destruction complex. We then examined if UCHL5 physically interacts with Axin1, which is a scaffolder of the ß-catenin destruction complex. Co-immunoprecipitation (Co-IP) analysis revealed that UCHL5 interacted with Axin1 in both ectopically and endogenously expressed conditions (Fig. 2c,d). In addition, confocal imaging showed that UCHL5 was partially co-located with the punctate form of Axin1 protein in the cytoplasm (Fig. 2e). In vitro binding assay using recombinant proteins demonstrated that UCHL5 directly interacts with Axin1 protein (Fig. 2f). We also confirmed that UCHL5 interacted with other components of the ß-catenin destruction complex, such as ß-catenin and GSK3ß (Fig. S1a, b). In order to understand the interaction mechanism in more detail, we performed Co-IP analyses using various deletion mutants of Axin1 (Fig. 2g). UCHL5 strongly bound to FL (full-length Axin1) and D2 (GSK3ß, ß-catenin, PP2A binding domain), whereas UCHL5 showed lower interaction with D1 (RGS) and failed to interact with D3 (DIX) (Fig. 2h). UCHL5 interacted with D4 (RGS; GSK3ß; ß-catenin), D5 (PP2A; DIX), and D6 (GSK3ß; ß-catenin), even though the interaction strength was variable (Fig. 2i). These results imply that the interaction between Axin1 and UCHL5 is most likely mediated by the coordination of multiple domains of Axin1 protein.

### UCHL5 stabilizes Axin1, but its deubiquitinating activity is dispensable

**Figure 3:**
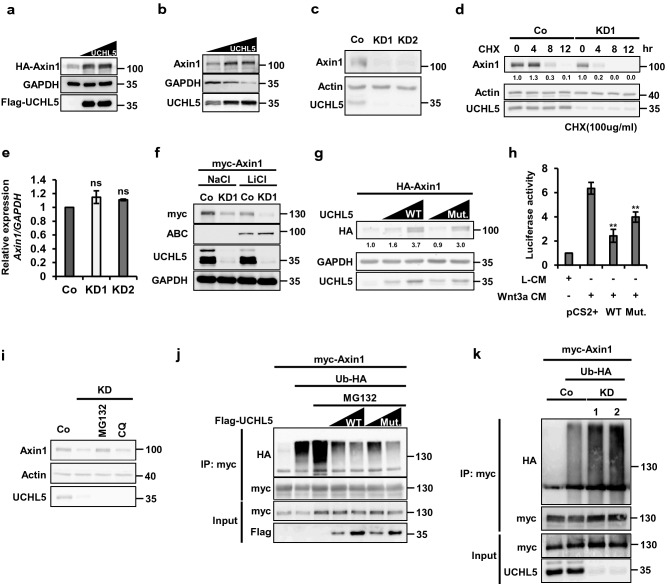
UCHL5 stabilizes Axin1 in a non-enzymatic fashion. (**a**) Western blot analysis using HeLa cells. Cells were transfected with HA-Axin1 (0.5 μg) and Flag-UCHL5 plasmids (2 μg and 3 μg). Cell lysates were then immunoblotted to detect the level of HA-Axin1, Flag-UCHL5, and GAPDH proteins. (**b**) Western blot analysis using HeLa cells. Flag-UCHL5 plasmids (2 μg and 3 μg) were introduced into cells. Cell lysates were then subjected to immunoblotting to detect endogenous Axin1, Flag-UCHL5, and GAPDH proteins. (**c**) Western blot analysis using Co, KD1, and KD2 HeLa cells. Endogenous Axin1, Actin, and UCHL5 levels were determined by anti-Axin1, anti-Actin, and anti-UCHL5. (**d**) Pulse-chase test using HeLa cells. The level of endogenous Axin1 protein was measured over time under the translation-blocked condition by cycloheximide (100 μg/ml). The band intensity of Axin1 was measured with ImageJ software and was normalized to Actin. (**e**) qPCR analysis for the expression of Axin1 mRNA in Co, KD1, and KD2 HeLa cells. The level of Axin1 mRNA was normalized by GAPDH mRNA. (**f**) Western blot analysis using Co and KD1 HeLa cells. Cells were transfected with myc-Axin1 (1 μg), and treated with either NaCl or LiCl (50 mM). Cell lysates were then subjected to immunoblotting to detect myc-Axin1, active ß-catenin, UCHL5, and GAPDH proteins. (**g**) Western blot analysis using HeLa cells. The requirement of deubiquitinating activity for the Axin1 stabilization was determined by comparing WT and Mut.UCHL5. HA-Axin1 (0.5 μg), WT UCHL5 (1 μg and 2 μg), and Mut. UCHL5 (1 μg and 2 μg) were introduced into KD1 HeLa cells. Cell lysates were immunoblotted with antibodies against HA, Actin, and UCHL5. The band intensity of HA-Axin1 was measured with ImageJ software and was normalized to GAPDH. (**h**) TOPflash assay using HeLa cells. Cells were transfected with TOPflash reporter (250 ng) and *TK-Renilla* reporter (50 ng), together with either WT UCHL5 or Mut.UCHL5. 8 h after transfection, cell culture media were replaced with Wnt3a CM and cells were further incubated for 16 h. (**i**) Western blot analysis using HeLa cells. KD1 HeLa cells were treated with either MG132 (20 μM) and Chloroquine (100 μM) for 5 h. Then, cell lysates were immunoblotted with antibodies for Axin1, UCHL5, and Actin. (**j**) In vivo ubiquitination assay using HeLa cells. myc-Axin1 (2 μg) was transfected alone or with Ub-HA (2 μg), together with either Flag-UCHL5 (2 μg and 4 μg) or Flag-Mut. UCHL5 (2 μg and 4 μg). After that, Cells were treated with MG132 (20 μM) for 5 h and then lysed and subjected to immunoprecipitation with anti-myc. (**k**) In vivo ubiquitination assay using HeLa cells. myc-Axin1 (2 μg) was transfected into WT and two KD HeLa cells (KD1 and KD2), alone or with Ub-HA (2 μg). Transfected cells were treated with MG132 (20 μM) for 5 h and then subjected to immunoprecipitation with anti-myc. The data from qPCR analysis (e) and TOPflash (h) and displayed as means ± SD and show a representative of multiple independent experiments (n = 3 biological independent experiments). ns, not significant and ** *P* < 0.005. from two-tailed unpaired t-test (**e**, **h**).

UCHL5 is a member of the Ubiquitin C-terminal hydrolase family, and it mediates deubiquitination of its substrate, thereby stabilizing target proteins. Therefore, we examined whether Axin1 is a substrate of UCHL5. On the one hand, the introduction of either Flag-UCHL5 or non-tagged UCHL5 into HeLa cells remarkably enhanced myc-Axin1 and endogenous Axin1 (Fig. 3a,b). On the other hand, the expression level of endogenous Axin1 decreased in UCHL5 KD cells (Fig. 3c). The pulse-chase test under translation-blocked condition clearly showed that the half-life of Axin1 was shortened by UCHL5 depletion (Fig. 3d). Furthermore, UCHL5 KDs did not alter the expression level of Axin1 mRNA (Fig. 3e). These data indicate that UCHL5 promotes the protein stability of Axin1. The reduction of Axin1 mediated by UCHL5KD was observed in both LiCl- treated and NaCl-treated conditions (Fig. 3f). This result implies that UCHL5 contributes to sustaining the physiological level of Axin1 protein. We also observed UCHL5 specifically regulated Axin1. UCHL5 KD did not change the expression level of endogenous APC and Axin2 (Fig. S2a), which are known as ubiquitin proteins.

Next, we determined the involvement of UCHL5 deubiquitinating activity in the regulation of Axin1 stability. To do this, we employed an enzymatically inactive mutant of UCHL5 (Mut.UCHL5). After the introduction of either wild-type UCHL5 (WT.UCHL5) or Mut.UCHL5 into UCHL5 KD cells, the effects of WT. UCHL5 and Mut.UCHL5 on Axin1 stabilization were compared. Interestingly, the protein level of HA-Axin1 was remarkably increased by both WT.UCHL5 and Mut.UCHL5 in a dose-dependent manner (Fig. 3g). Consistently, both WT and Mut.UCHL5 significantly blocked TOPFlash activity (Fig. 3h, p < 0.005). These unexpected results suggest that UCHL5 mediates the stabilization of Axin1 protein regardless of its deubiquitinating activity. It has been well-known that proteins are degraded through either the proteasome- or the lysosome-dependent pathway. We thus asked which mechanism is involved in UCHL5-mediated Axin1 stabilization. To this end, we conducted a rescue assay with two different inhibitors, MG132 (20 μM), a proteasome inhibitor, and chloroquine (CQ, 1 μM), a lysosome inhibitor. Notably, MG132 rescued the UCHL5KD-dependent reduction of Axin1, whereas CQ did not (Fig. 3i). The data demonstrated that UCHL5 stabilizes Axin1 by blocking proteasome-dependent degradation. It is known that the proteasome complex degrades proteins labeled with ubiquitin moieties, leading us to assume that UCHL5 has a function in blocking the ubiquitination of Axin1 protein. However, experimental results revealed that the deubiquitinating activity of UCHL5 is dispensable for this process. Indeed, both WT.UCHL5 and Mut.UCHL5 led to decreased ubiquitination of Axin1 protein in a dose dependent manner (Fig. 3j). In contrast, ubiquitination further increased in UCHL5 KD cells (Fig. 3k). The results imply two important aspects of UCHL5 in reference to the regulation of Axin1 protein. First, Axin1 is constantly exposed to an unknown protein degradation pathway mediated by ubiquitination. Second, UCHL5 is essential to sustain the proper level of Axin1 by blocking ubiquitination rather than removing ubiquitin moieties from Axin1 protein. Indeed, in vitro ubiquitination assay showed that recombinant UCHL5 protein did not remove the ubiquitin moieties from the Axin1 protein (Fig. S2b).

### UCHL5 mediates Axin1 stabilization through the DIX domain

**Figure 4:**
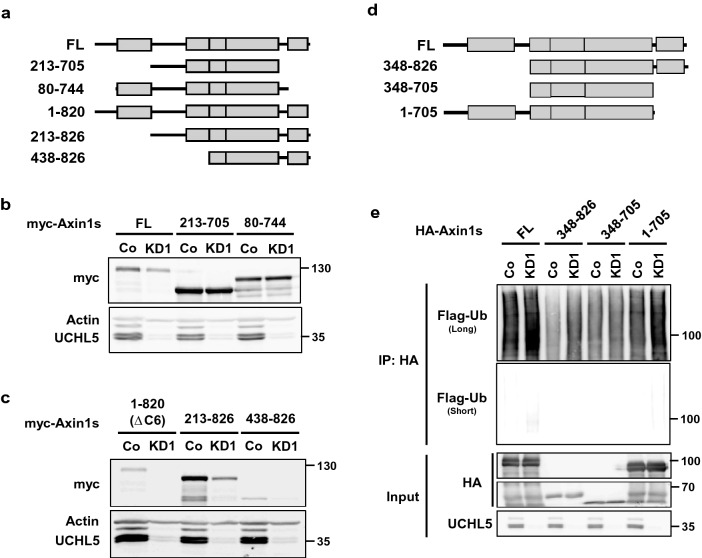
DIX domain is crucial for UCHL5-mediated Axin1 stabilization. (**a**) Diagram of a series of Axin1 deletion mutants used for western blotting. (**b**,**c**) 1 μg of myc- full-length Axin1 (FL) and truncated Axin1 mutants (213–705 a.a., 80–744 a.a., 1–820 a.a., 213–826 a.a., 438–826 a.a.) were transfected into WT and KD1 HeLa cells. Resulting cells lysates were subjected to immunoblotting with antibodies against myc, Axin, and UCHL5. (**d**) Diagram of a series of Axin1 deletion mutants used for ubiquitination assay. (**e**) In vivo ubiquitination assay using Co and KD1 HeLa cells. HA-Axin1 (2 μg) and Ub-Flag (2 μg) were transfected into WT and two KD1 HeLa cells. The transfected cells were treated with MG132 (20 μM) for 5 h and then subjected to immunoprecipitation with anti-HA.

In order to understand the mechanism of Axin1 stabilization by UCHL5, we first determined the responsive element of Axin1 to be degraded when UCHL5 is depleted. For this analysis, we constructed various fragments of Axin1 protein (Fig. 4a, Fig. S4). Depletion of UCHL5 led to a remarkable reduction of Full-length, 1–820, 213–826, 438–826, and 348–826 fragments (Fig. 4b,c, Fig. S4). However, UCHL5 KD had no effect on the 1–705, 213–705, and 80–744 fragments (Fig. 4b, Fig. S4). This result implies that the C-terminal region of Axin1, including the DIX domain, may be crucially involved in UCHL5-mediated Axin1 stabilization. Given that UCHL5 can block the ubiquitination of Axin1 for its stabilization, we speculated that the ubiquitination associated with UCHL5 would arise in the DIX domain by an unknown E3 ligase. To verify this, we performed an in vivo ubiquitination assay using the truncated Axin1 constructs (Fig. 4d,e). Interestingly, UCHL5 KD enhanced the poly-ubiquitination of Full-length, 348–826 and 1–705 fragments, whereas UCHL5 KD did not change the level of poly-ubiquitination of 348–705 fragment (Fig. 4e). This result supports the hypothesis of UCHL5 suppressing ubiquitination in the DIX domain of Axin1, although UCHL5 regulates both N-and C-terminal ends of Axin1.

### UCHL5 is necessary for Axin1 polymerization

**Figure 5:**
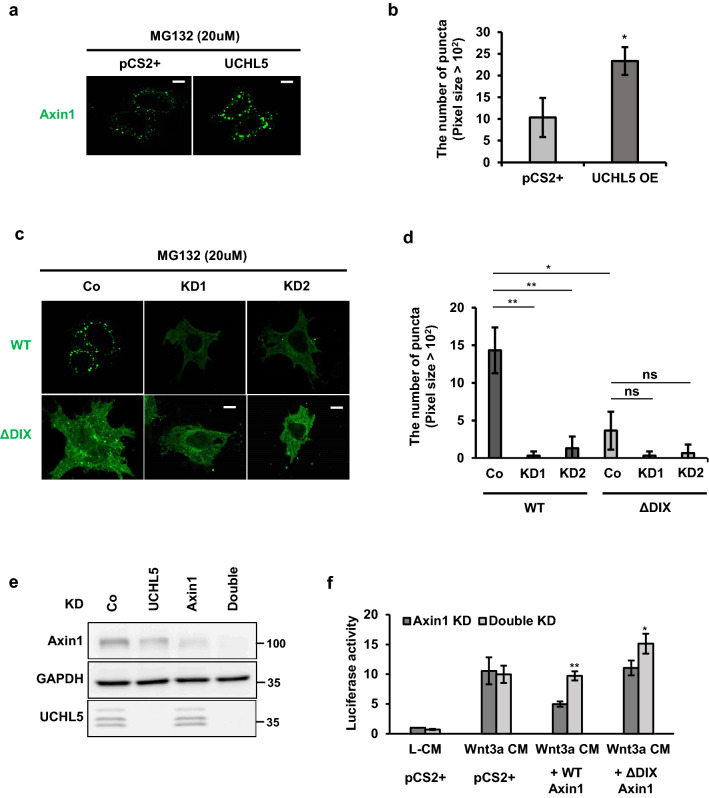
UCHL5 is required for DIX-mediated polymerization of Axin1. (**a**) Immunocytochemistry assay. GFP-Axin1 plasmids (400 ng) were introduced into cells with either empty vector (2 μg) or UCHL5 plasmids (2 μg). The transfected cells were treated with MG132 (5 μM) for 16 h. Then, the cells were fixed and immunostained with anti-GFP and Alexa 488 mouse secondary antibody. Scale bars represent 10 μm. (**b**) Quantification of the GFP puncta average number from the images in (**a**) performed using ImageJ quantification tool. Quantification performed from 3 experiments with 5 cells quantified for each condition. Data are displayed as means ± SD and show a representative of three independent experiments. *P < 0.05 from two-tailed *t*-test. (**c**) Immunocytochemistry assay. GFP-Axin1 plasmids (400 ng) were introduced into WT and KD cells (KD1 and KD2). Then, the transfected cells were treated with MG132 (5 μM) for 16 h. After that, the cells were fixed and immunostained as described in a. Scale bars represent 10 μm. (**d**) Quantification of the GFP puncta average number from the images in (**c**) performed using ImageJ quantification tool. Quantification performed from 3 experiments with 5 cells quantified for each condition. Data are displayed as means ± SD and show a representative of three independent experiments. ns, not significant. *P < 0.05, **P < 0.005, from two-tailed *t*-test. (**e**) Western blot analysis using HeLa cells. Lysates from Axin1-deficient (Axin1 KD) and Axin1/UCHL5-double deficient cells (Double KD) were immunoblotted with anti-Axin1, anti-UCHL5, and anti-GAPDH. (**f**) TOPflash assay using HeLa cells. Either Axin1 KD or Double KD HeLa cells were transfected with TOPflash reporter (250 ng) and *TK-Renilla* reporter (50 ng), together with either wild-type Axin1 (500 ng) or DIX-deficient Axin1 (ΔDIX; 500 ng). 8 h after transfection, cell culture media were changed with Wnt3a CM and cells were further incubated for 16 h. The data is displayed as means ± SD and show a representative of multiple independent experiments (n = 3 biological independent experiments). *P < 0.05, **P < 0.005.

It is well known that a key feature of DIX proteins is their ability of homo- and hetero-interaction, with themselves and other DIX proteins, respectively^13^. Along the same lines, two DIX proteins in the Wnt signaling cascade, Axin and Dvl, can also form protein complex through either the hetero- or homo-interaction, and regulates the Wnt signaling pathway differently according to its interacting partners^19^. For signaling activation, Dvl forms a hetero-complex with Axin through their DIX domains. This complex is a prerequisite for Axin recruitment to the plasma membrane^13^. In contrast, the homo-polymerization of Axin is essential for efficient assembly of the ß-catenin destruction complex and ß-catenin turnover. This Axin polymerization is also accomplished through DIX-mediated head to tail interaction^19^. Therefore, we verified the involvement of UCHL5 in this DIX-mediated Axin polymerization through an immunostaining analysis. As previously reported, Axin1 formed polymers in the transfected cells, called puncta, which are more visible in cells where UCHL5 is co-expressed (Fig. 5a,b). Conclusively, Axin1 showed the faint and diffuse expression in UCHL5 KD cells (Fig. 5c,d), whereas DIX-deficient Axin1 showed a diffuse pattern regardless of UCHL5 expression (Fig. 5c,d). To elaborate on the direct effect of UCHL5 KD-dependent depolymerization on Wnt signal activity, we constructed single knockdown HeLa cells for Axin1, and double knockdown HeLa cells for Axin1 and UCHL5 to avoid the influence of endogenous Axin1 in Axin1 polymerization (Fig. 5e). We then performed TOPFlash analysis and compared luciferase activities between single and double knockdown cells (Fig. 5f). In order to verify the effect of UCHL5 on the DIX domain, either WT Axin1 or DIX-deficient Axin1 was reintroduced into single and double knockdown cells. The treatment of Wnt3a CM comparably enhanced luciferase activity in both single and double knockdown cells (Fig. 5f), which clearly supported that UCHL5 is functionally involved in Axin1-mediated Wnt regulation. The reintroduction of WT. Axin1 significantly suppressed the luciferase activity in single knockdown cells, while showing lower suppression activity in double knockdown cells (Fig. 5f). Notably, in contrast to WT. Axin1, DIX-deficient Axin1 failed to suppress the luciferase activity in single knockdown cells and showed slightly enhanced activity in double knockdown cells (Fig. 5f). Our results clearly demonstrate that UCHL5 is required for DIX-mediated Axin1 polymerization, thereby enhancing the functional activity of the ß-catenin destruction complex to downregulate Wnt signaling activity.

## Discussion

Axin is a cytosolic scaffolder protein and a core component of the ß-catenin destruction complex^20^. Since the expression level of Axin protein is extremely low compared to other components in the ß-catenin destruction complex, it is working as a concentration-limiting factor^6^. With this reason, cellular responses to Wnt stimulation are sensitively dependent on both the concentration and functional activity of Axin protein^5,6,12^. It has been known that the physiological activity of Axin is determined by its concentration, structural changes, and inter-intra molecular interactions^6,14,19^. Therefore, understanding how these events are regulated is an essential step to gain comprehensive and deep insight into the ß-catenin turnover mechanism. There are two paralogs of Axin proteins, Axin1 and Axin2/Conductin^14^. Even though they are functionally redundant, recent studies have demonstrated that Axin2 has a lower suppressive activity on Wnt signaling than Axin1^14^. Thus, we can speculate that Axin1 likely plays a more dominant role in the suppression of Wnt signaling activity. Therefore, discovering molecules that regulate Axin1 is essential to understanding the suppression mechanism of Wnt signaling in normal conditions, making it a promising therapeutic target for Wnt-related diseases.

In this study, we revealed that UCHL5 is a novel component of the ß-catenin destruction complex, specifically required for protein stability and functional activity of Axin1. The perturbation of UCHL5 expression in cells promoted the destabilization of Axin1 protein and disrupted DIX-mediated polymerization. The protein concentration of Axin is mainly regulated by ubiquitination and the proteasome-dependent pathway^11^. Indeed, Axin is known as one of the ubiquitinating proteins. Ubiquitin-mediated Axin degradation is achieved by two representative E3 ligases, SIAH1/2 and RNF146^6–8^. They mediate Axin degradation in a Wnt- and PARylation-dependent manner, respectively^6,8^. However, even though these E3 ligases strongly dictate the concentration of Axin, these E3 ligase-mediated Axin degradations are only confirmed under artificial conditions. Indeed, in the normal state, Axin sustains its constant protein level inside the cell, and it is only perturbed when the Wnt signal is ectopically activated or post-translational modifications, such as ubiquitination and PARylation, are enforced by either genetic engineering or treatment of specific inhibitors. Furthermore, Wnt signal activation far precedes Axin degradation. For instance, ß-catenin begins to stabilize as early as 30 min following Wnt stimulation. However, significant reduction of Axin protein begins at a later time (at 4 h post-Wnt stimulation)^21^. These facts strongly suggest the existence of a protective mechanism to maintain Axin’s physiological level by blocking protein degradation caused by either Wnt-dependent or independent degrative mechanism. Thus, deubiquitinating enzymes would be more important determinants of Axin concentration than E3 ligases. In this regard, two ubiquitin-specific proteases have been reported. First, USP34 can rescue Axin1 from PARylation-dependent degradation^10^. However, USP34 mediates the nuclear localization of Axin1, and it positively regulates Wnt signaling pathway. Second, a recent genetic screening using CRISPR/CAS9 method revealed USP7 as a negative regulator of Wnt signaling pathway as a deubiquitinase for Axin^11^. USP7 interacts with Axin1 and removes ubiquitin moieties conjugated by both SIAH1/2 and RNF146. Importantly, our study demonstrates that there is not only a protective mechanism by deubiquitinating enzymes, but also an alternative protection mechanism for Axin1, which has not been known to date. For instance, even though UCHL5 is also classified as a deubiquitinating enzyme, its deubiquitinating activity is dispensable for Axin1 stabilization. Consistently, Wild-type and catalytically inactive UCHL5 comparably suppressed the activity of Wnt signaling pathway, and had almost a similar efficacy on Axin1 stabilization. Interestingly, this nonenzymatic effect of UCHL5 on Axin1 stabilization is clearly achieved by protecting Axin1 from ubiquitination. Our ubiquitination assay results showed that Axin1 was ubiquitinated less under the UCHL5-overexpressed condition. In contrast, the ubiquitination of Axin1 was enhanced under the UCHL5-depleted condition. However, UCHL5 recombinant proteins did not remove polyubiquitination of Axin1. These results suggest that UCHL5 may physically block an Axin1-targeting E3 ligase unknown to this study. Notably, we suppose that this mechanism is independent of SIAH1/2 and Tankyrase/RNF146, since the depletion of UCHL5 still led to the destabilization of Axin1 mutants (VxP mutants), which have a resistance against SIAH1/2-mediated degradation (Fig. S3a, b). In addition, IWR-1, Tankyrase inhibitor failed to rescue the Axin1 destabilization induced by UCHL5 depletion (Fig. S3c). Furthermore, we observed that the DIX domain is crucial for UCHL5-associated Axin1 stabilization and ubiquitination. Given that Tankyrase/RNF146 targets the N-terminus of Axin1 and SIAH1/2 competes with GSK3ß over the GSK3ß-binding domain^6–8^, UCHL5 likely protects Axin1 from another E3 ligase. Therefore, the identification of the E3 ligase responsible for Axin degradation is necessary in future studies.

As for the functional activity of Axin, its DIX-dependent polymerization is necessary^13^. Many previous studies have shown that DIX-domain deficiency or mutation led to defects in polymerization, as well as the failure of its suppression activity of Axin^13^. The depletion of UCHL5 also results in a defect in Axin1 polymerization and the loss of suppression activity of Axin1. Our comparison study using WT. Axin1 and DIX-deficient Axin1 revealed that these UCHL5-associated defects affect the DIX domain. Moreover, our observation that the ubiquitination sites associated with UCHL5 involve the DIX domain supports our understanding that ubiquitination events possibly attenuate DIX-mediated polymerization of Axin1. Although the involvement of ubiquitination in Axin polymerization has not yet been reported, a recent interesting study showed the involvement of ubiquitination in DIX-mediated polymerization of Dvl. Dvl DIX domain is ubiquitinated at two lysines (K54 and K58), and ubiquitination at K54 blocks DIX-dependent polymerization by interfering with the DIX-DIX interface^22^. We think that UCHL5-associated ubiquitination in Axin1 DIX domain may also work in the same way to disrupt Axin1 polymerization. However, the molecular mechanism of DIX ubiquitination blockage by UCHL5 remains ambiguous. Intriguingly, our results indicate that UCHL5 blocks DIX ubiquitination without interacting with the DIX domain. Therefore, this is likely to require Axin1’s dynamic conformational changes in consequence of its intra-molecular interactions. These intra-molecular interactions are regulated by either GSK3ß-mediated phosphorylation or Wnt signal activation^5,19,23^. Coincidentally, UCHL5 interacts with Axin1 through multiple domains (1–705; RGS, GSK3ß, ß-catenin, and PP2A binding domain) where intra-molecular interactions arise. In all likelihood, these multiple interactions allow UCHL5 to protect the DIX domain from ubiquitination at all times despite the dynamic conformational change of Axin1. Furthermore, the depletion of UCHL5 also led to the additional ubiquitination in N-terminal end of Axin1, which may suggest another functional layer of UCHL5 even though it is not related to the stability of Axin1 and not addressed in this study. Therefore, future studies based on protein structure and post-translational modifications must be conducted to understand the exact molecular mechanism.

Another interesting part catching our attention was that the altered expression of UCHL5 (OE or KD) only showed a significant change in Wnt signal activity under the Wnt-active condition. We presume the physiological role of UCHL5 is to protect ß-catenin destruction complex from its inhibition by Wnt-activation, thereby keeping a certain threshold level of signal responsiveness against Wnt stimulation. That way, the biological environment (cell or tissue) can stably secure Wnt-off state. Hence, UCHL5 KD or its impaired function gets the environment sensitized to Wnt stimulation.

Our study provides important insight on UCHL5 as a novel negative regulator of Wnt signaling pathway. Previously, we reported UCHL5 as a positive regulator of Wnt signaling pathway in *Xenopus* embryos and HepG2 liver cancer cell^24^. In another recent study using endometrial cancer cells, UCHL5 reportedly promotes cancer growth by positively regulating the Wnt signaling pathway^25^. Interestingly, our current study using HeLa, MCF7, SW480, and A549 showed a negative effect of UCHL5 on Wnt signaling pathway (Fig. S5a, d). These observations imply that UCHL5 regulates Wnt signaling pathway differently in accordance with the biological context.

It is a widely accepted fact that abnormal Wnt signal activation leads to hyper-proliferation, metastasis, and invasion^26^. However, despite the negative effect of UCHL5 on Wnt signaling pathway in cancer cells, we failed to observe more aggressive cancer progression caused by hyperactivation of Wnt signaling pathway under the UCHL5-depleted condition (Fig. S5b, c). UCHL5 knockdown suppressed cancer cell growth even though it promoted Wnt signal activity (Fig. S5d, e). In previous studies, the inhibition of UCHL5 by b-AP15 or its loss of function causes caspases-dependent apoptosis, a consequence of the endoplasmic reticulum stress response related to the decline of proteasome activity^27,28^. Although the knockdown effect of UCHL5 on the promotion of cancer activity is hidden by proteotoxic stress, the effect of the DIX ubiquitination antagonized by UCHL5 on Wnt-related cancers should be examined in a future study. To do this, information on an E3 ligase is an essential prerequisite.

**Figure 6:**
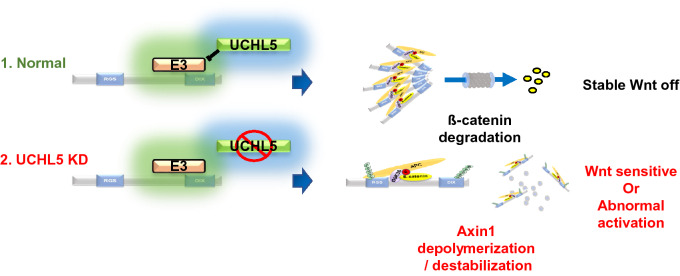
UCHL5 is required for the functional activity of Axin1. UCHL5 blocks ubiquitination of Axin1 DIX domain. As a result, Axin1 is stabilized and successfully polymerized. However, absence of UCHL5 promotes DIX ubiquitination and results in failure of Axin1 polymerization and stabilization. The image was generated using Microsoft PowerPoint software Version 1907 (Build 11901.20176).

In summary, our results suggest that UCHL5 acts as a negative regulator of the Wnt signaling pathway. Furthermore, this study provided the opportunity to advance our understanding of UCHL5 functions within the ß-catenin destruction complex, where it helps maintain the functional activity of Axin1 by promoting both protein stability and polymerization of Axin1 (Fig. 6). The detailed mechanism study demonstrates that UCHL5 protects Axin1 DIX domain from ubiquitination. The regulation mechanism of Axin protein is complex and sophisticated. As for the ubiquitination of Axin, it has been exclusively studied from the perspective of protein stability. However, ubiquitination governs protein functions in many different ways, such as protein stability, molecular interaction, localization, and protein structure^29^. In that sense, this study also suggests a new mechanistic insight into the versatile role of ubiquitination in Axin1 regulation.

## Materials and methods

### Plasmids

UCHL5 was amplified from cDNA of HEK293T by PCR and inserted into pCS2 + (XhoI/XbaI), pCS4-6Flag (BamH1/ClaI) or pCS4-3HA (BamH1/ClaI) vector. Catalytically inactive UCHL5 (Mut.UCHL5) was previously described and generated by PCR-directed mutagenesis^24^. Axin1 constructs were kindly provided by Dr. Frank Costantini. All truncated mutants and point mutations of Axin1 were generated by PCR and then inserted into pCS2 + 6myc (Xho1/Xba1), pCS4-HA (XhoI/XbaI), and pEBG (Cla/Not1). pCS2 + ubiquitin-HA and pCS-ubiquitin-Flag were kindly provided by Dr. Eek-Hoon Jho. GSK3 and ß-catenin were PCR-amplified from cDNA of HEK293T and inserted into pCS4-3HA and pCS2 + GFP, respectively.

### Cell culture, RNA interference, and transfection

HeLa, MCF7, SW480, and A549 cells (ATCC) were maintained in DMEM High glucose (Hyclone) supplemented with 10% FBS (Hyclone) and 1% penicillin–streptomycin (Sigma P0781) at 37 °C with 5% CO_2_.

Stable KD HeLa cells expressing short hairpin RNA (shRNA) for UCHL5 depletion were generated by infection of lentiviral package. Two independent shRNA sequences for UCHL5 were: 5′-TCCCGACTTGACACGATATTT-3′ and.

### 5′-AGCCAGTTCATGGGTTAATTT-3′

All plasmids were transfected using Lipofectamin 2000 (Invitrogen), according to the manufacturer’s protocol.

### Luciferase reporter assay

Cells were seeded in a 24 well plate. 250 ng of TOPFlash and 50 ng of Renilla were transfected the following day. 24 h after transfection, cells were lysed and subjected to luciferase reporter assay using Dual-Luciferase® Reporter Assay System (Promega), according to the manufacturer’s protocol. Duplicates were repeated three times independently, and average figures were displayed with standard deviation (SD). P-value was calculated using two-tailed test, * *P* < 0.05, ** *P* < 0.005.

### cDNA synthesis and qPCR analysis

The extraction of total RNA was performed using TRIZOL reagent (Invitrogen) according to the manufacturer's protocol (https://www.thermofisher.com /trizol.html; TRIzol User Guide). For cDNA synthesis, 1ug of total RNA was reacted with M-MLV reverse transcriptase (Promega) and oligo(dT) 15 primer according to the manufacturer's protocol (https://www.promega.com › Resources › Protocols).

We performed quantitative real-time PCR (qPCR) analysis as described before^24^. The synthesized cDNA was analyzed using the StepOne real-time PCR system (Applied Biosystems). PCR conditions were 95 °C (10 min) and 40 cycles of 95 °C (30 s) and 60 °C (1 min). Data are displayed as mean with SD from triplicates. *P* value was calculated using two-tailed test, *P < 0.05, **P < 0.005.

Primers used were:

**Table:** 

Genes	Primer sequences (5′ → 3′)
Axin2	TTA TGC TTT GCA CTA CGT CCC TCC A
	CGC AAC ATG GTC AAC CCT CAG AC
c-Myc	CCT GGT GCT CCA TGA GGA GAC
	CAG ACT CTG ACC TTT TGC CAG G
UCHL5	TGT GGT TCA GGA CTC CCG ACT T
	CGC CTA AAT GGA CAT CCT GGT G
Axin1	GTA TGT GCA GGA GGT TAT GCG G
	CAC CTT CCT CTG CGA TCT TGT C

### Western blot, immunoprecipitation analysis, in vitro binding assay, ubiquitination assay

For western blot analysis, cells were harvested in cold PBS and lysed in RIPA buffer (20 mM Tris–Cl, pH 7.5, 1% Triton X-100, 0.1% SDS, 150 mM NaCl, 1 mM EDTA, 1 mM EGTA, 1 mM β-glycerophosphate, 1 mM Na3VO4, and protease inhibitor cocktail). Samples were cleared by centrifugation and then separated on SDS-PAGE gels, followed by transfer to PVDF or nitrocellulose membrane at 100 V for 2 h at 4 °C. Western blot images were acquired using LAS-4000 system (FujiFilm).

For immunoprecipitation analysis, cells were lysed in modified RIPA buffer (20 mM Tris–Cl, pH 7.5, 1% NP40, 150 mM NaCl, 1 mM EDTA, 1 mM EGTA, 1 mM β-glycerophosphate, 1 mM Na3VO4, and protease inhibitor cocktail). 1 mg of lysates were incubated with antibodies and Protein A-Sepharose® 4B. After that, immunoprecipitates were subjected to western blot analysis.

For in vitro binding assay, 3 μg of GST or GST-Axin1 (SignalChem) was incubated with UCHL5 (R&D system) in modified RIPA buffer at 4 °C for 6 h. Glutathione-Sepharose beads (Peptron) were added to pulldown the protein complexes. After that, beads were washed four times using IP buffer and then, samples were analyzed by western blot analysis.

For ubiquitination assay, cells were incubated with 20 μM MG132 (Sigma) for 5 h. After that, cells were rinsed with cold PBS twice and boiled in SDS lysis buffer (20 mM Tris–Cl, pH 7.5, 1% SDS, 150 mM NaCl, 1 mM DTT) at 100 °C for 5 min. the boiled samples were diluted 10 times with modified RIPA buffer, and then the samples were chilled at 4 °C for 1 h. After that, samples were subjected to immunoprecipitation and western blot analysis sequentially.

We performed in vitro ubiquitination assay as described before^24^. For in vitro ubiquitination analysis, both myc-Axin1 and Flag-Ub were transfected to HeLa cells. After 48 h, cells were lysed in IP buffer. Cell lysates were centrifuged and then, supernatants were sequentially incubated with indicated antibody and Protein G sepharose (Invitrogen) at 4 °C for 3 h. Beads were washed 3 times with in vitro DUB buffer (50 mM Tris–Cl (pH7.5), 150 mM NaCl, 1 mM EDTA and 1 mM DTT). Washed beads were incubated with indicated amount of UCHL5 recombinant proteins (R&D system) at 37 °C for 2 h in 50 μl of in vitro DUB buffer. The reaction was terminated by adding SDS sample buffer and subsequent boiling at 95 °C for 10 min. Samples were analyzed by western blot analysis.

### Immunofluorescence

Cells were plated on coverslips and cultured in 12 well plates. 48 h after transfection, cells were washed with PBS and fixed in 4% PFA for 5 min at room temperature. For immunostaining, cells were permeabilized with 0.2% Triton X-100 in PBS for 5 min, followed by blocking with 10% goat-serum for 1 h. After that, cells were incubated with primary antibodies (1:200) overnight, and secondary antibodies (Alexa 488 and 594, Invitrogen,1:500) for 2-5 h. The stained cells were mounted in UltraCruz® Aqueous Mounting Medium with DAPI (Santa Cruz). Images were acquired using FV3000 confocal laser scanning microscope (Olympus). The images were quantified using the default ‘Analyze particles’ plugin in ImageJ.

### Statistics

A minimum of three independent replicates were performed for each experiment. The error bars shown represent the standard deviation (SD) of at least three independent experiments, Statistical relevance was determined using a two-tailed Student’s *t*-test. Asterisks (*) indicate statistical significance (* *P* < 0.05, ** *P* < 0.005, *** *P* < 0.0005).

## Supplementary Information

Supplementary Figures.
