# Supplementary material for: UCHL5 controls β-catenin destruction complex function through Axin1 regulation
Source: Sci Rep. 2022 Mar 7;12:3687. doi: 10.1038/s41598-022-07642-1 (PMC8901634; doi:10.1038/s41598-022-07642-1)
Supplement: Supplementary file 1 — Supplementary Figures. [file 41598_2022_7642_MOESM1_ESM.pdf]

Supplementary Information

9 Supplementary figures with legends

UCHL5 controls ß-catenin destruction complex function through Axin1 regulation

Wonhee Han[1,#], Youngmu Koo[2,#], Leila Chaieb[2], Byeong-Rak Keum[2], Jin-Kwan Han[2,*]

1. F. M. Kirby Neurobiology Center, Boston Children’s Hospital, Department of Neurology, Harvard Medical School, Boston, MA 02115, USA.

2. Department of Life Sciences, Pohang University of Science and Technology, 77 Cheongam-Ro, Nam-Gu, Pohang, Gyeongbuk, 37673, Korea.

# Contributed equally

* Corresponding author: jkh@postech.ac.kr

Figure S1

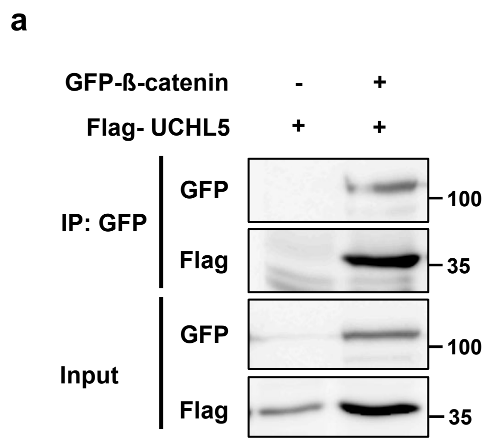
a
GFP-ß-catenin - +
Flag- UCHL5 + +
IP: GFP
GFP
Flag
Input
GFP
Flag
100
35
100
35

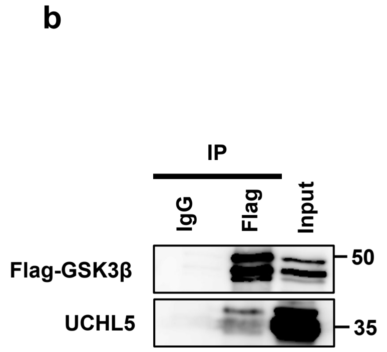
b
IP
IgG
Flag
Input
Flag-GSK3β
UCHL5
50
35

Figure S2

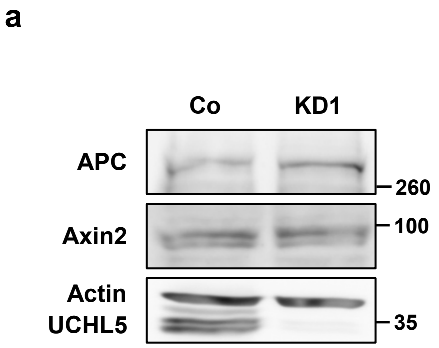

a
Co
KD1
APC
260
Axin2
100
Actin
UCHL5
35

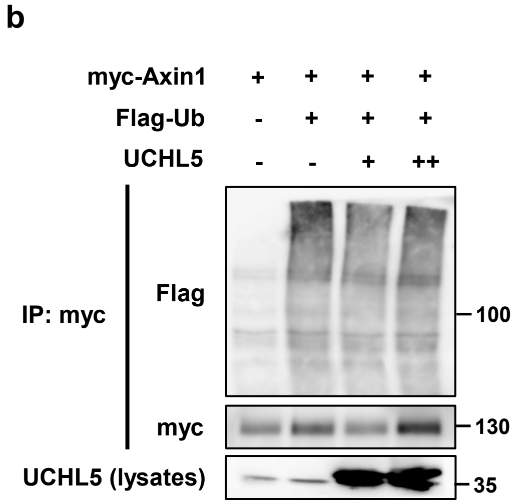

b
myc-Axin1 + + + +
Flag-Ub - + + +
UCHL5 - - + ++
IP: myc
Flag
100
myc
130
UCHL5 (lysates)
35

Figure S3

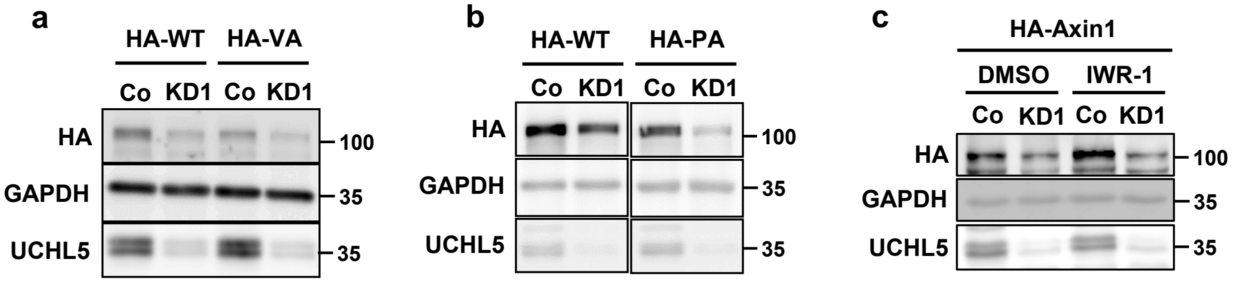

a
HA-WT
HA-VA
Co
KD1
Co
KD1
HA
100
GAPDH
35
UCHL5
35
b
HA-WT
HA-PA
Co
KD1
Co
KD1
HA
100
GAPDH
35
UCHL5
35
c
HA-Axin1
DMSO
IWR-1
Co
KD1
Co
KD1
HA
100
GAPDH
35
UCHL5
35

Figure S4

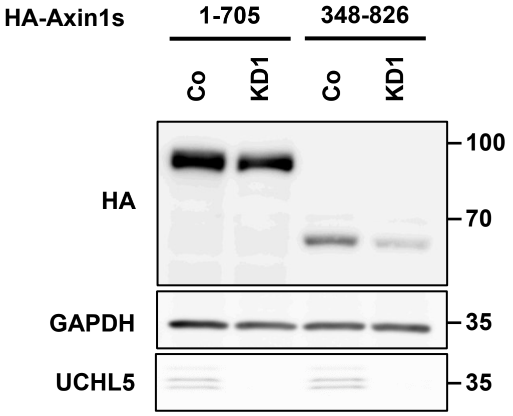

Figure S5

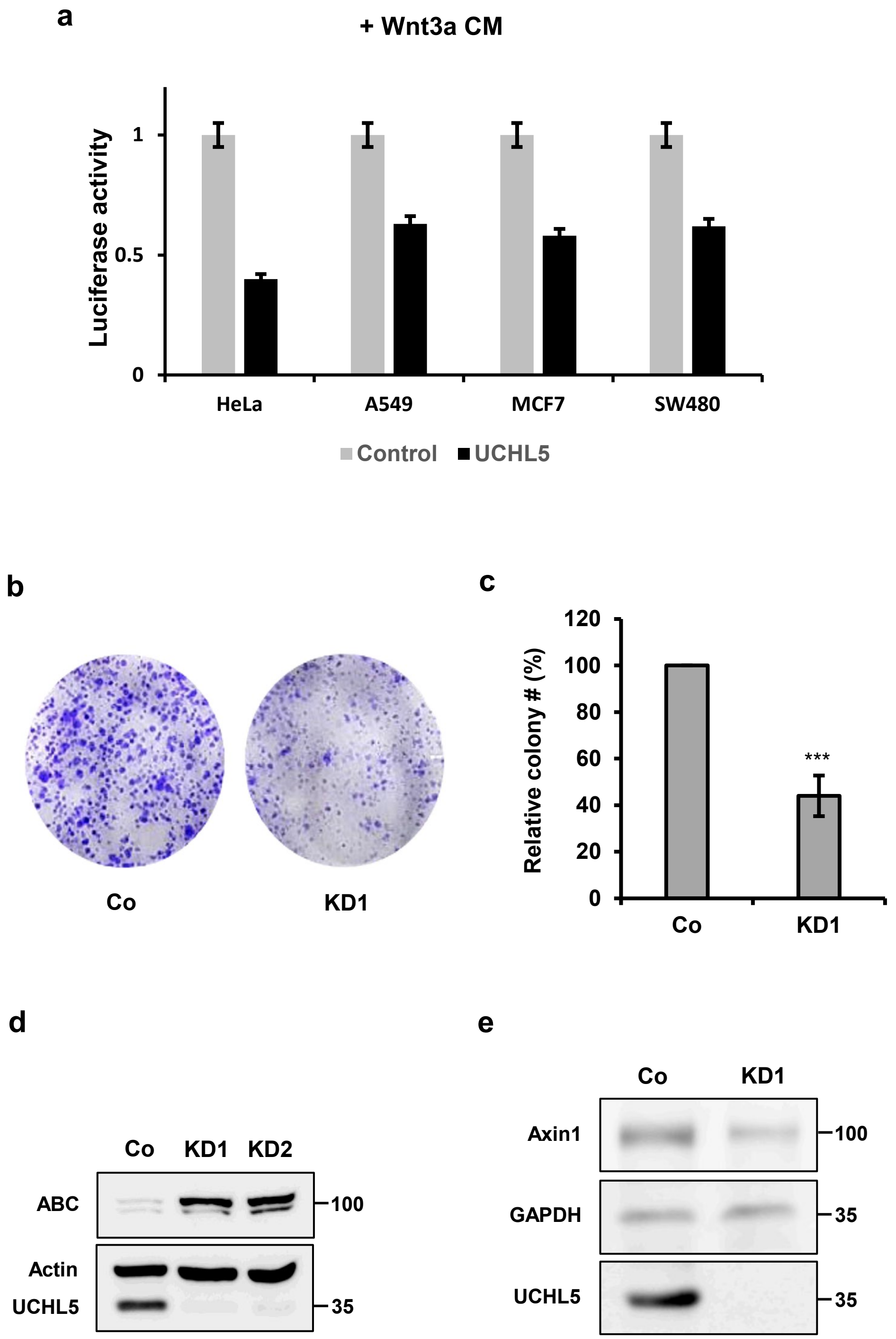
a
+ Wnt3a CM
Luciferase activity
1
0.5
0
HeLa
A549
MCF7
SW480
Control
UCHL5
b
Co
KD1
c
Relative colony # (%)
120
100
80
60
40
20
0
***
Co
KD1
d
Co
KD1
KD2
ABC
100
Actin
UCHL5
35
e
Co
KD1
Axin1
100
GAPDH
35
UCHL5
35

Figure S6

Figure 1c

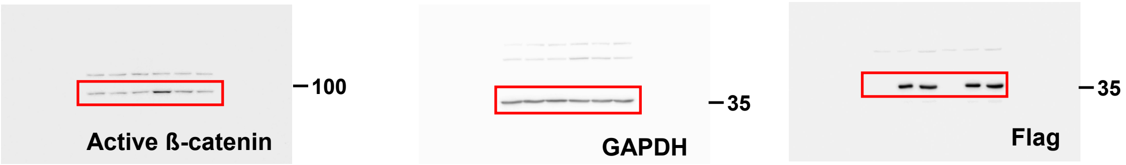

Figure 1g

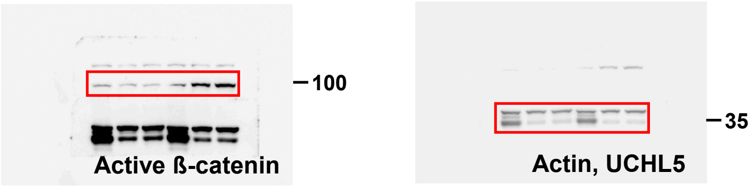

Figure 2b

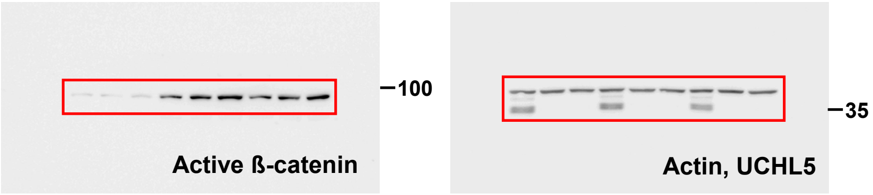

Figure 2c

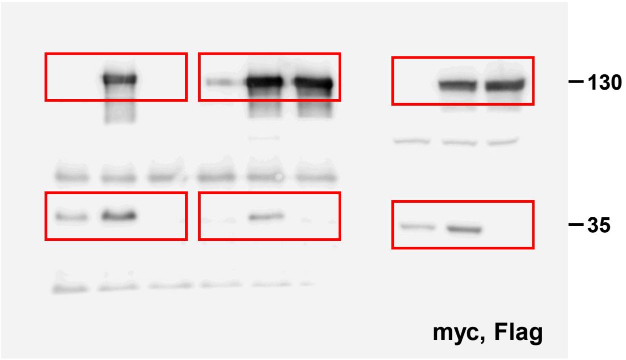

Figure 2d

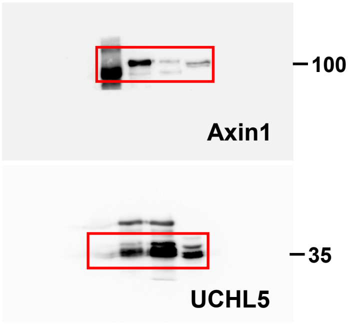

Figure 2f

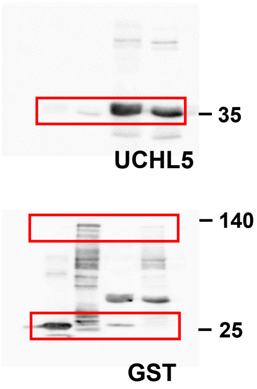

Figure 2h

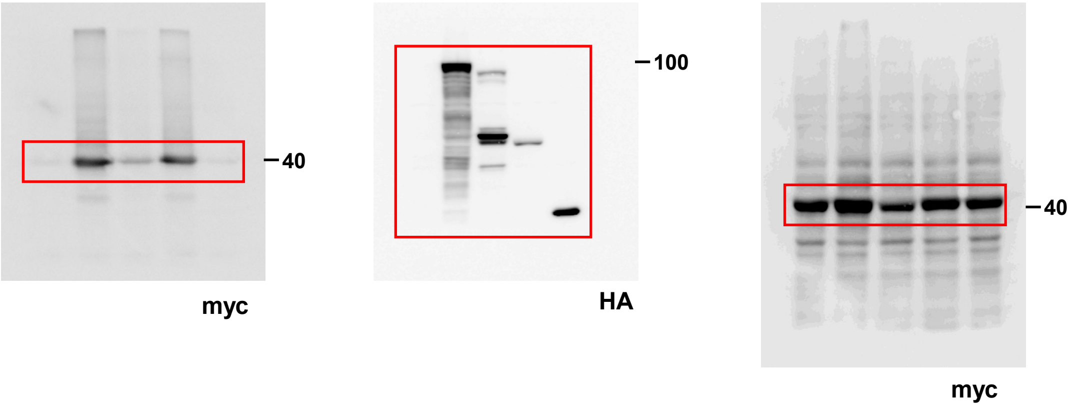

Figure S7

Figure 2i

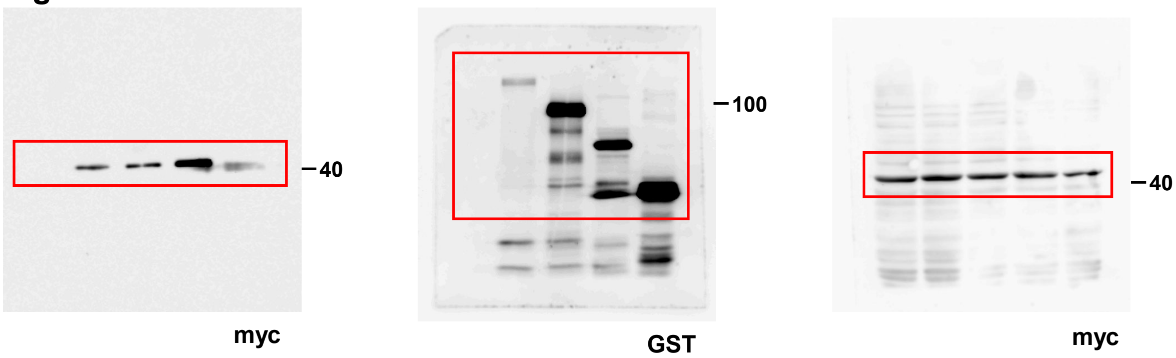

Figure 3a

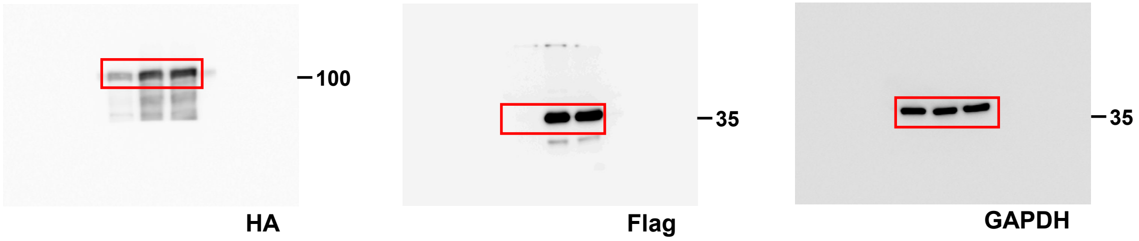

Figure 3b

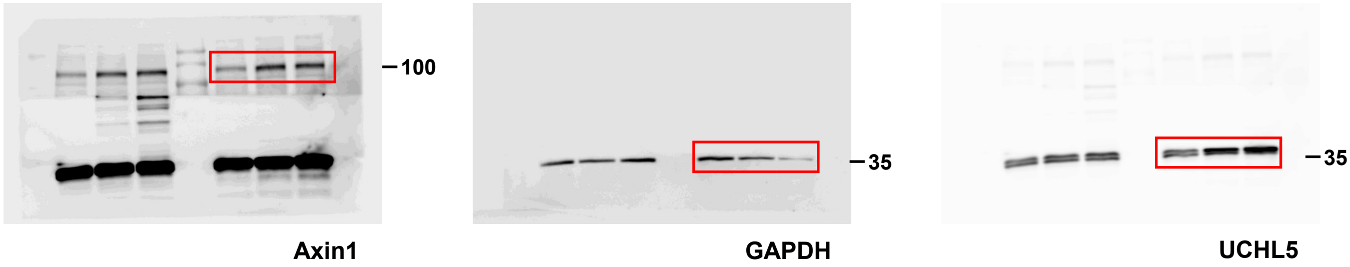

Figure 3c

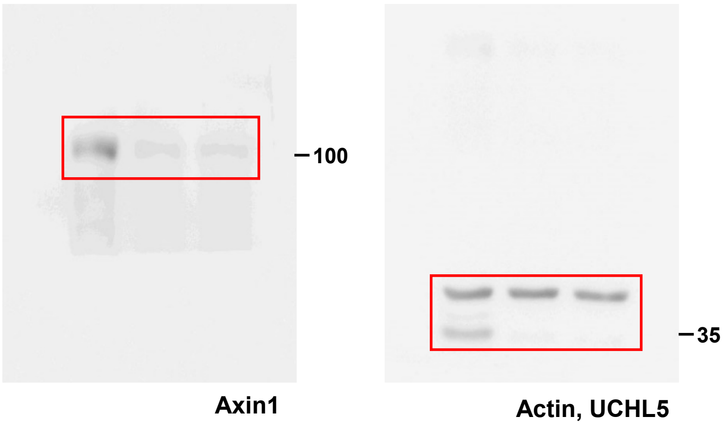

Figure 3d

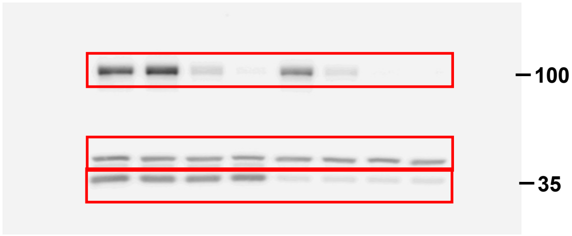

Figure S8

Figure 3f

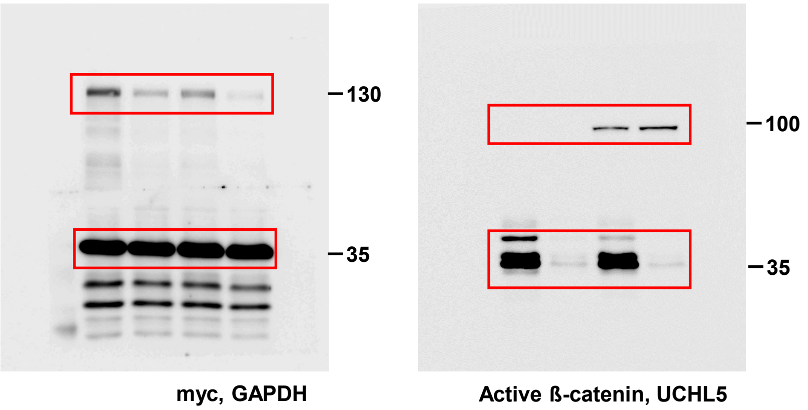

Figure 3g

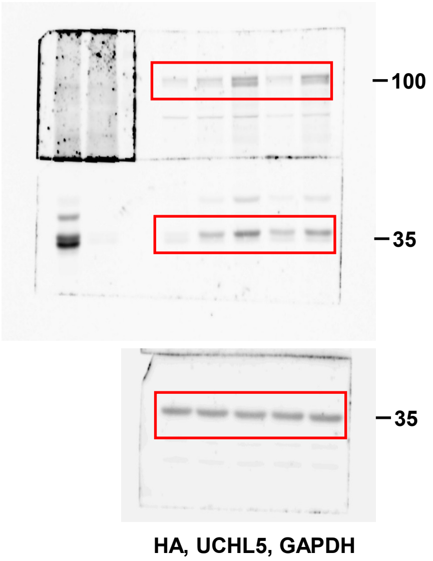

Figure 3i

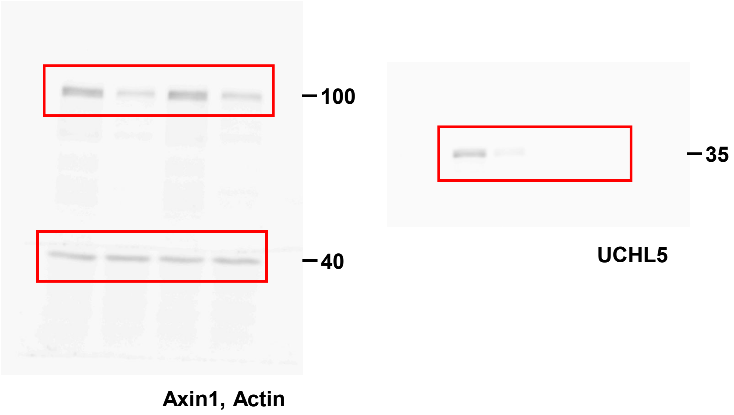

Figure 3j

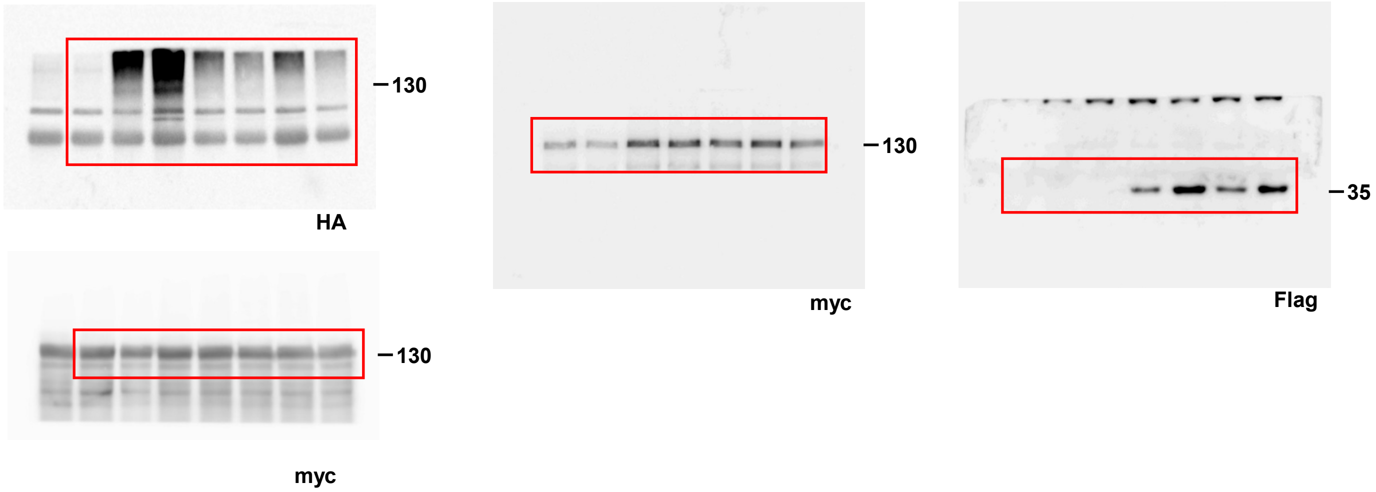

Figure S9

Figure 3k

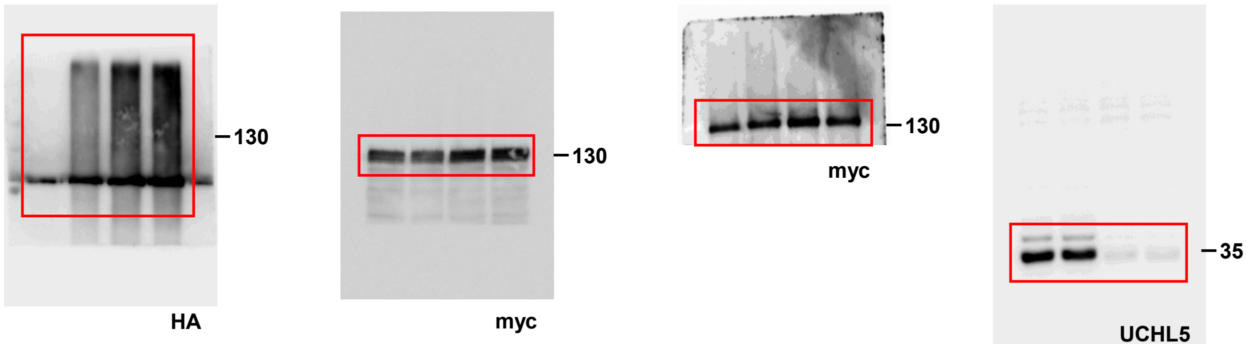

Figure 4b

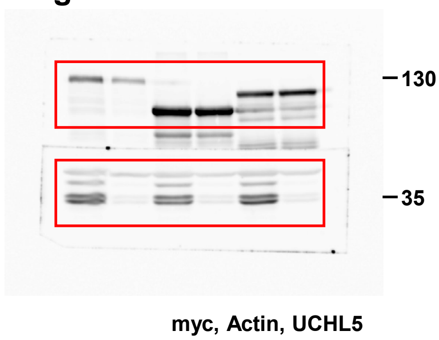

Figure 4c

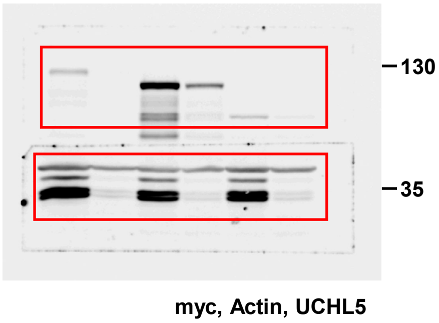

Figure 4e

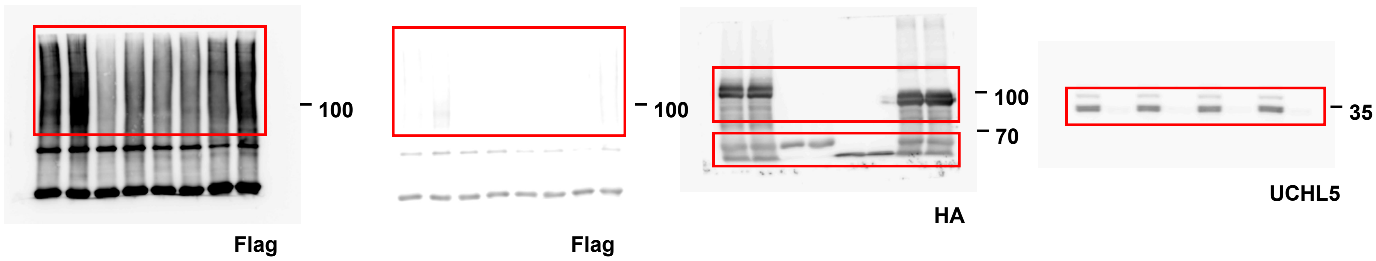

Figure 5c

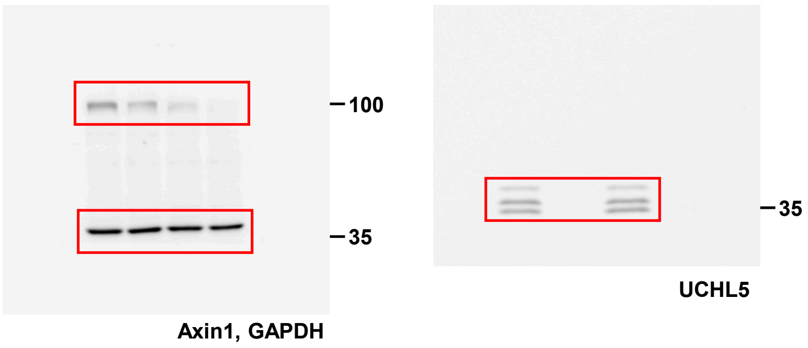

Figure S1. UCHL5 interacts with components of ß-catenin destruction complex. a. IP assay using HeLa cells. Cells were transfected with Flag-UCHL5 (2μg) and GFP-ß-catenin (2μg). Then, cell lysates were subjected to immunoprecipitation with anti-GFP. **b.** IP assay using HeLa cells. Cells were transfected with Flag-GSK3ß (2μg). Then, cell lysates were subjected to immunoprecipitation with anti-Flag.

Figure S2. UCHL5 stabilizes Axin1, but its deubiquitinating activity is dispensable. a. Western blot analysis using Co and KD1 HeLa cells. Cell lysates were immunoblotted with endogenous APC, Axin2, Actin, and UCHL5 antibodies. **b.** *In vitro* ubiquitination assay. HeLa cells were co-transfected with both myc-Axin1 and Flag-Ub plasmids to express polyubiquitinated Axin1. After 48 h, cell lysates were immunoprecipitated using anti-myc antibody to obtain polyubiquitinated Axin1. Precipitated polyubiqutinated Axin1 was then incubated with UCHL5 recombinant proteins (+ 1ug; ++ 2ug).

Figure S3. UCHL5 stabilizes Axin1 in an independent mechanism from SIAH1/2 and Tankyrase. a, b. Western blot analysis using Co and KD1 HeLa cells. Cells were transfected with 0.5μg of Wild type Axin1 and two Axin1VxP mutants (HA-WT, HA-VA, and HA-PA). Cell lysates were then immunoblotted to detect the level of HA-Axin1, UCHL5, and GAPDH proteins. **c.** Western blot analysis using Co and KD1 HeLa cells. Cells were transfected with 0.5μg of HA-Axin1, then treated with either DMSO or IWR-1(12Mm) for 16hrs. Cell lysates were immunoblotted to detect the level of HA-Axin1, UCHL5, and GAPDH proteins.

Figure S4. UCHL5 mediates Axin1 stabilization through the DIX domain. 1μg of truncated Axin1 mutants (1-705 a.a., 348-826 a.a.) were transfected into WT and KD1 HeLa cells. Resulting cells lysates were subjected to immunoblotting with antibodies against HA, GAPDH, and UCHL5.

Figure S5. UCHL5 negatively regulates Wnt signal activity in various cancer cells. a. TOPflash assay using HeLa, A549, MCF7, and SW480. Cells were transfected with the indicated plasmids (50ng *TK-Renilla* reporter; 250ng TOPFlash; 250ng FOPFlash; 2μg pCS2+UCHL5). Then, Wnt3a CM was treated for 16hrs. **b, c.** Clonogenic assay and qualitative analysis of SW480 cells . 200 cells were seeded into a 6-well plate and cultured for 2 weeks until each cell formed colony. colonies were then fixed and stained with crystal violet (*** $p < 0.0005$). The data are presented as the mean ± SD of the experiments performed in triplicate. . **d, e.** Western blot analysis using Co and KD SW480 cells. Cell lysates were immunoblotted to detect the level of active β-catenin, Axin1, UCHL5, Actin, and GAPDH proteins

Figure S6-S9. Full-length images of gels and blots from the main figures.
